# Protective Effects of Lactoferrin against SARS-CoV-2 Infection In Vitro

**DOI:** 10.3390/nu13020328

**Published:** 2021-01-23

**Authors:** Claudio Salaris, Melania Scarpa, Marina Elli, Alice Bertolini, Simone Guglielmetti, Fabrizio Pregliasco, Corrado Blandizzi, Paola Brun, Ignazio Castagliuolo

**Affiliations:** 1 Department of Molecular Medicine, University of Padua, 35121 Padua, Italy; claudio.salaris@studenti.unipd.it (C.S.); alice.bertolini@studenti.unipd.it (A.B.); ignazio.castagliuolo@unipd.it (I.C.); 2 Laboratory of Advanced Translational Research, Veneto Institute of Oncology IOV-IRCCS, 35128 Padua, Italy; melania.scarpa@iov.veneto.it; 3 AAT-Advanced Analytical Technologies S.r.l., Fiorenzuola d’Arda, 29122 Piacenza, Italy; marina.elli@aat-taa.eu; 4 Department of Food, Environmental and Nutritional Sciences (DeFENS), University of Milan, 20122 Milan, Italy; simone.guglielmetti@unimi.it; 5 IRCCS Istituto Ortopedico Galeazzi, University of Milan, 20136 Milan, Italy; fabrizio.pregliasco@unimi.it; 6 Unit of Pharmacology and Pharmacovigilance, Department of Clinical and Experimental Medicine, University of Pisa, 56126 Pisa, Italy; corrado.blandizzi@med.unipi.it

**Keywords:** lactoferrin, SARS-CoV-2, antiviral immunity, COVID-19

## Abstract

SARS-CoV-2 is a newly emerging virus that currently lacks curative treatments. Lactoferrin (LF) is a naturally occurring non-toxic glycoprotein with broad-spectrum antiviral, immunomodulatory and anti-inflammatory effects. In this study, we assessed the potential of LF in the prevention of SARS-CoV-2 infection in vitro. Antiviral immune response gene expression was analyzed by qRT-PCR in uninfected Caco-2 intestinal epithelial cells treated with LF. An infection assay for SARS-CoV-2 was performed in Caco-2 cells treated or not with LF. SARS-CoV-2 titer was determined by qRT-PCR, plaque assay and immunostaining. Inflammatory and anti-inflammatory cytokine production was determined by qRT-PCR. LF significantly induced the expression of *IFNA1*, *IFNB1*, *TLR3*, *TLR7*, *IRF3*, *IRF7* and *MAVS* genes. Furthermore, LF partially inhibited SARS-CoV-2 infection and replication in Caco-2 intestinal epithelial cells. Our in vitro data support LF as an immune modulator of the antiviral immune response with moderate effects against SARS-CoV-2 infection.

## 1. Introduction

A novel coronavirus has generated a pandemic outbreak, designated as COVID-19, that was first reported in Wuhan, China, 2019 and spread rapidly all over the world [1]. The 2019 coronavirus is a single-stranded RNA virus, designated as SARS-CoV-2, as it has genetic similarities with SARS-CoV, the virus responsible for the severe acute respiratory syndrome outbreak that occurred in 2002 [2]. The genome of coronaviruses encodes several proteins, including spike (S), thought to initiate the infection, envelope (E), membrane (M) and nucleocapsid (N). The cellular entry of SARS-CoV-2 has been shown to depend on the binding of the viral spike glycoprotein (SgP) to host cell angiotensin converting enzyme 2 (ACE-2) and host cell surface heparan sulfate proteoglycans (HSPGs) [3,4,5].

SARS-CoV-2 is primarily thought to infect the lungs with transmission through respiratory droplets (aerosols), mainly during close person-to-person contact [6]. Moreover, the intestine represents another viral target organ supporting SARS-CoV-2 replication [7], with viral shedding into stool occurring in a substantial portion of patients, which makes fecal-oral transmission also plausible [8]. Effective therapeutic options against SARS-CoV-2 are still under development. Currently, therapeutic interventions against SARS-Cov-2 infection rely on supportive care and respiratory support for severely ill people as well as some antivirals [9,10], and drugs targeted against the abnormal immune-inflammatory response [10,11].

The development of novel antiviral drugs requires considerable time and efforts. Therefore, options based on natural products could represent reliable and safe integrative tools against COVID-19. Lactoferrin (LF), a glycoprotein belonging to the transferrin family, naturally present in various human secretions, is known to bind and transport iron and to play an important role in regulating iron homeostasis [12]. In-vitro studies on human intestinal, hepatic and T cell lines showed that LF has an interesting potential in fighting viral and bacterial infections and to exert anti-inflammatory and immune-modulating activity [13,14,15]. Many studies have proven that LF can exert significant effects against viruses, including hepatitis C virus, herpes simplex virus, human immunodeficiency virus, poliovirus and rotavirus, with in vitro EC_50_ values (half maximal effective concentration) generally in the micromolar range [16,17]. The LF antiviral effect generally occurs in the early phase of infection, preventing the entry of viral particles into the host cells, either by directly binding to the viral particles or blocking the virus receptor or co-receptor present on the host cell [18]. Notably, LF is able to prevent the internalization of some viruses, among which SARS pseudovirus [13], by binding to cell-surface HSPGs, which have been shown to act as a necessary co-factors also for SARS-CoV-2 infection [5,19]. Moreover, LF was shown to inhibit the entry of murine coronavirus [20], as well as human coronaviruses hCoV-NL63 [21], that are closely related to SARS-CoV-2. LF was also reported to selectively inhibit cathepsin L [22], a lysosomal peptidase critical for endocytosis, which is a cell entry pathway used by SARS-CoV-2 [23,24]. Another major aspect of LF bioactivity relates to its immunomodulatory and anti-inflammatory actions [25]. Indeed, LF was demonstrated to reduce interleukin 6 (IL6) and tumor necrosis factor-alpha (TNFα) in experimental settings simulating sepsis [26]. Thus, it is possible that LF may modulate the overactive immune and inflammatory response to SARS-CoV-2 that in some patients leads to acute respiratory distress and subsequent mortality [27]. Overall, LF could represent a non-toxic health supplement to prevent infection as well as an adjunct treatment for those who have developed COVID-19 [28].

In vitro cell models for SARS-CoV-2 research are essential for understanding the viral life cycle, for amplifying and isolating the virus for further research and for preclinical evaluation of therapeutic molecules [29]. Promising preliminary evidence on the immunomodulatory properties of this LF under study were obtained by testing the glycoprotein on intestinal cell lines prior to the first Italian COVID-19 outbreak. In this study, we used the intestinal epithelial Caco-2 cell line, expressing ACE2 receptor, to investigate the effects of LF on antiviral immune response gene expression and SARS-CoV-2 infection in vitro.

## 2. Materials and Methods

### 2.1. Cells, Virus, and Reagents

Human colon adenocarcinoma cell line Caco-2 (ATCC HTB-37, ATCC, Manassas, VA, USA) and monkey kidney epithelial cell line Vero E6 (ATCC CRL-1586, ATCC, Manassas, VA, USA) were grown in Dulbecco’s Modified Eagle’s Medium (DMEM) supplemented with 10% (*v*/*v*) FBS, 1% (*v*/*v*) sodium pyruvate and 1% (*v*/*v*) penicillin/streptomycin (all from Gibco-Thermo Fisher Scientific, Waltham, MA, USA) at 37 °C in humidified incubator containing 5% CO_2_. SARS-CoV-2 was isolated from a patient at the Microbiology Unit, University Hospital of Padua. The viral strain was propagated in Vero E6 cells and characterized by whole genome sequencing. LF (Globoferrina/Transferrin, bovine lactoferrin, SOFAR SpA, Trezzano Rosa, MI, Italy) was resuspended in antibiotic-free medium and used at a final concentration of 100 µg/mL.

### 2.2. Viral Stocks Preparation and Titration

Viral titer was determined by the plaque assay method. Briefly, confluent VERO E6 cells in 24-well plates (Costar, Merck, KGaA, Darmstadt, Germany) were inoculated with serial 10-fold dilutions of the virus stock for 1 h. Then, culture medium was removed and cells incubated with fresh medium containing carboxymethylcellulose (CMC, Merck KGaA, Darmstadt, Germany). Cells were fixed 72 h post-infection with 5% *w*/*v* formaldehyde (Merck KGaA, Darmstadt, Germany) and stained with crystal violet (Merck KGaA, Darmstadt, Germany). Virus titer was measured as plaque-forming units (PFU/mL) based on the plaques formed in cell culture upon infection.

All the infection experiments were performed in a biosafety level 3 (BSL-3) laboratory at the Department of Molecular Medicine, University of Padua, Padua, Italy.

### 2.3. Caco-2 Cell Culture and Lactoferrin Treatments

**Figure 1:**
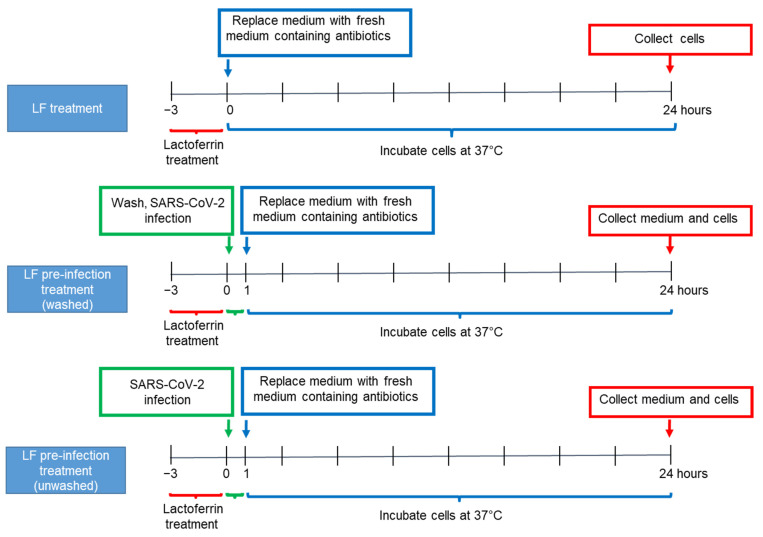
Experimental design. Lactoferrin treatments and SARS-CoV-2 infection of Caco-2 cell line. LF: Lactoferrin.

Caco-2 cells were seeded in 12-well plates (2 × 10^5^ cells/mL). After reaching confluence, cells were washed in Phosphate-buffered saline (PBS),Gibco-Thermo Fisher Scientific, Waltham, USA) and incubated in antibiotic-free medium (AFM) (untreated control) or subjected to one of the treatments described below, according to the experimental design displayed in Figure 1. In all treatment protocols, Caco-2 confluent cells were supplemented with LF at a concentration of 100 μg/mL for 3 h. Then, in the LF treatment group of experiments, cells were washed in PBS (Gibco-Thermo Fisher Scientific, Waltham, MA, USA) and incubated with fresh medium supplemented with antibiotics (penicillin/streptomycin). Cells were harvested 24 h later for RNA extraction. In the LF pre-infection treatment (washed) protocol, cells were washed in PBS (Gibco-Thermo Fisher Scientific, Waltham, MA, USA), supplemented with fresh medium with antibiotics (penicillin/streptomycin) and infected with SARS-CoV-2 (multiplicity of infection (MOI) 2:1) for 1 h. Twenty-four hours post-infection, cells were harvested for RNA extraction and supernatants for viral titration. In the LF pre-infection treatment (unwashed) group, cells were infected with SARS-CoV-2 (MOI 2:1), without washing away LF. After 1 h, cells were washed and incubated with fresh medium supplemented with antibiotics (penicillin/streptomycin) for further 24 h before harvesting for RNA extraction and supernatants viral titration.

### 2.4. RNA Extraction and Real-Time RT-PCR

Total RNA was isolated using the E.Z.N.A. Total RNA Kit I (Omega Bio-Tek Inc., Norcross, GA, USA) following the manufacturer’s instructions. Contaminating DNA was removed by incubation with RNase-free DNase I set (Omega Bio-Tek Inc., Norcross, GA, USA). Complementary DNA synthesis and amplification were performed using the iTaqTM Universal Probes One-Step Kit (Bio-Rad, Milan, Italy) according to the manufacturer’s directions in an ABI PRISM 7000 Sequence Detection System (Applied Biosystems, Foster City, CA, USA). The cycling conditions were: 50 °C for 10 min, 95 °C for 3 min, and 40 cycles at 95 °C for 15 s and 60 °C for 60 s. The expression of the target gene was normalized to the expression of the GAPDH housekeeping gene. The specific forward and reverse primers used are summarized in Table 1 and were designed with Primer-BLAST software. *RdRp IP2* and *CoV E* primers specific for SARS-CoV-2 detection were taken from the Pasteur Institute and Charitè protocol, respectively [30]. Data are presented as a mean fold change over the control.

### 2.5. Immunofluorescence

Caco-2 cells were seeded in sterile coverslips inside 6-well plates. After reaching confluence, cells were subject to one of the previously described treatment protocols and infected with SARS-CoV-2 at MOI 2:1. Twenty-four hours post-infection cells were fixed with 4% paraformaldehyde, blocked with 2.5% bovine serum albumin (BSA) in TBS 1x buffer containing 0.5% Triton for 1 h at room temperature. The cells were washed and incubated with the primary antibody (SARS-CoV-2 spike antibody, GeneTex GTX135360) for 1 h at room temperature, followed by incubation with the secondary antibody (Alexa Fluor 488 goat anti-rabbit IgG, Life Technologies, Carlsbad, CA, USA) for 1 h at room temperature. Viral protein expression was visualized using confocal microscopy (Nikon A1).

### 2.6. Statistical Analysis

All experiments were performed in duplicate wells for each condition and repeated at least three times. Data are shown as mean +/− SD. Statistical analysis was performed using GraphPad Prism Software 6.0 (GraphPad Software Inc., La Jolla, CA, USA). Comparisons were performed using two-tailed Student’s t test. Differences were considered significant at *p* < 0.05.

## 3. Results

### 3.1. Lactoferrin Enhances the Antiviral Immune Response in Uninfected Caco-2 Intestinal Epithelial Cells

**Figure 2:**
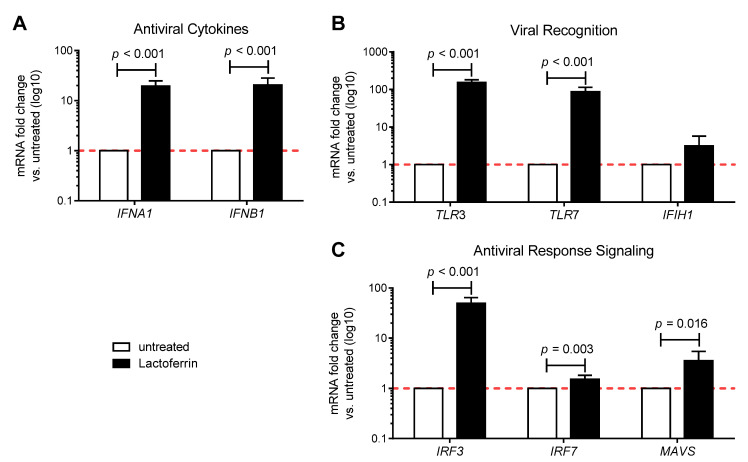
Lactoferrin enhances the antiviral immune response in vitro. Gene expression of (**A**) type I interferons, (**B**) innate immune receptors and (**C**) regulatory molecules of innate immune response was assessed by real-time qPCR in Caco-2 cells 24 h after a 3-h treatment with 100 µg/mL lactoferrin (n = 3–7). Data are shown as relative fold changes compared to untreated (arbitrarily set as 1) and presented as mean + SD.

The antiviral immunomodulatory effects of LF were tested In vitro using human intestinal epithelial Caco-2 cells. Confluent cells were supplemented with 100 μg/mL LF for 3 h and antiviral immune gene expression was analyzed 24 h post initial treatment. The transcript levels of both antiviral cytokines interferon (IFN) alpha (*IFNA1*) and beta (*IFNB1*) were significantly enhanced by LF (Figure 2A). Furthermore, LF treatment could also induce the mRNA expression of toll-like receptor 3 (*TLR3*) and 7 (*TLR7*), which are pattern recognition receptors involved in sensing of RNA viruses (Figure 2B), and of interferon regulatory factor 3 (*IRF3*) and 7 (*IRF7*), as well as mitochondrial antiviral-signaling (*MAVS*), which participate to antiviral innate immunity response signaling pathways (Figure 2C). LF also led to an increase, although not significant, in the expression of interferon induced with helicase C domain 1 (*IFIH1*), which encodes a cytoplasmic receptor critical for sensing of RNA viruses (Figure 2B). Overall, these results suggest that LF has antiviral immunomodulatory properties in vitro.

### 3.2. Lactoferrin Partially Inhibits SARS-CoV-2 Infection in Caco-2 Intestinal Epithelial Cells

**Figure 3:**
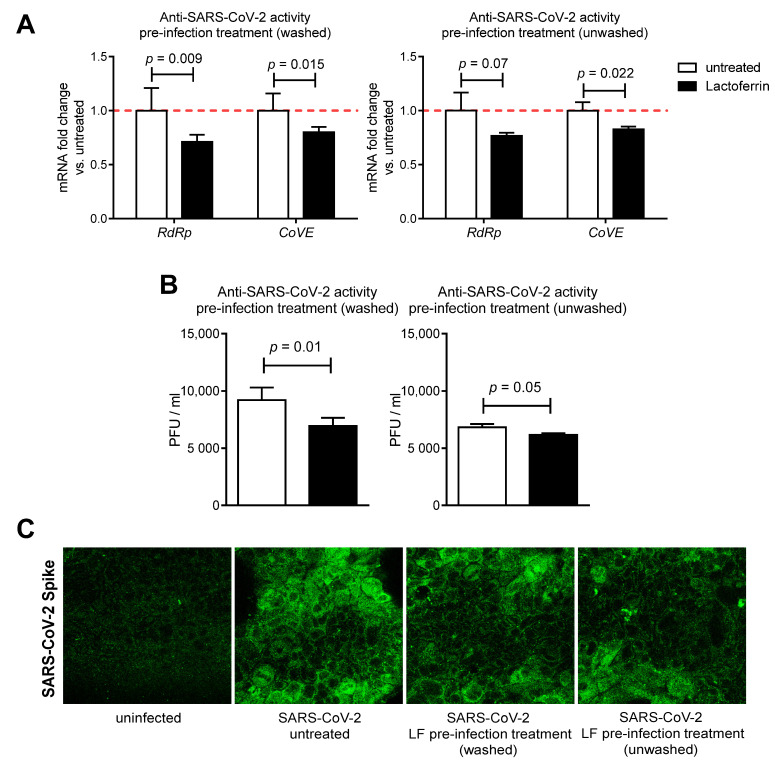
Lactoferrin protects intestinal epithelial cells from SARS-CoV-2 infection in vitro. (**A**) Caco-2 cells were treated or not for 3 h with 100 µg/mL lactoferrin and then infected with SARS-CoV-2 according to experimental design shown in Figure 1. SARS-CoV-2- specific gene expression was assessed by real-time qPCR 24 h post infection (n = 3–6). Data are shown as relative fold changes compared to untreated (arbitrarily set as 1). (**B**) SARS-CoV-2 titer was determined by plaque assay performed on harvested supernatants (n = 4). Data are presented as mean + SD. (**C**) Representative staining for SARS-CoV-2 spike of infected Caco-2 cells pre-treated or not with lactoferrin according to experimental design shown in Figure 1.

To evaluate the antiviral activity of LF against SARS-CoV-2, an infection assay for SARS-CoV-2 was performed in Caco-2 cells. The expression level of the virus-specific gene coding RNA-dependent RNA polymerase (*RdRp*) and E gene (*CoVE*), critical for SARS-CoV-2 replication and assembly, was analyzed from total RNA obtained from harvested cells 24 h post infection. As shown in Figure 3A, the expression of *RdRp* gene was significantly reduced in LF-pre-treated (washed) infected cells and tended to be lower in LF-pre-treated (unwashed) cells, as compared to untreated infected Caco-2 cells. Furthermore, *CoVE* gene expression was significantly decreased by both LF pre-infection washed and unwashed treatments. Notably, SARS-CoV-2 titer of the harvested supernatants revealed that LF pre-infection treatments (washed and unwashed) resulted in 24%and 7% inhibition of SARS-CoV-2 infection, respectively (Figure 3B). Moreover, immunofluorescence staining of Caco-2 infected cells for the viral spike glycoprotein confirmed that both LF pre-infection washed and unwashed treatments decreased SARS-CoV-2 infection (Figure 3C). These data suggest that LF pre-infection washed and unwashed treatments partially inhibit SARS-CoV-2 infection and replication in Caco-2 intestinal epithelial cells.

### 3.3. Lactoferrin Pre-Infection Treatments Modulate Cytokines Production Triggered by SARS-CoV-2 in Caco-2 Intestinal Epithelial Cells

**Figure 4:**
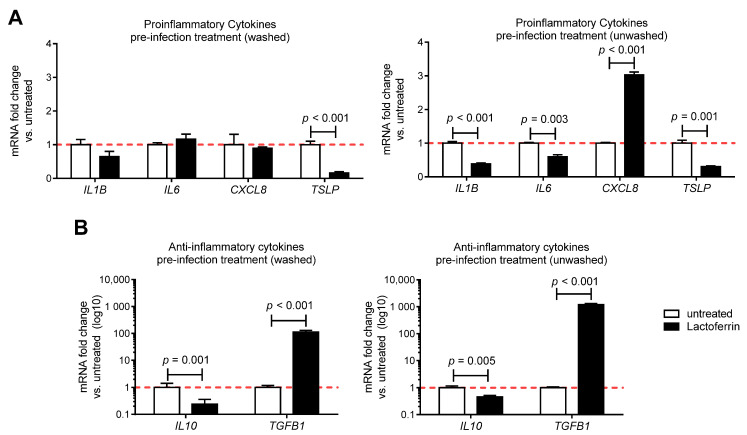
Lactoferrin modulates cytokine production resulting from SARS-CoV-2 infection in vitro. Caco-2 cells were treated or not for 3 h with 100 µg/mL lactoferrin and then infected with SARS-CoV-2 according to experimental design shown in Figure 1. Gene expression of (**A**) proinflammatory cytokines and (**B**) anti-inflammatory cytokines was assessed by real-time qPCR 24 h post infection (n = 3–6). Data are shown as relative fold changes compared to untreated (arbitrarily set as 1) and presented as mean + SD.

To determine whether LF pre-infection treatments could dampen the inflammatory response triggered by SARS-CoV-2 infection in vitro, the expression profile of inflammatory and anti-inflammatory cytokines of SARS-CoV-2 infected-Caco2 cells pre-treated or not with LF was tested (Figure 4). The transcript levels of all measured cytokines were significantly upregulated following infection with SARS-CoV-2 (Supplementary Figure S1). Notably, pre-treatments of infected Caco-2 cells with LF significantly reduced the transcript levels of thymic stromal lymphopoietin (*TSLP*) long isoform, an upstream epithelial Th2 cytokine, known to exert multiple pathogenic effects (Figure 4A). LF pre-infection treatment (unwashed) also significantly decreased the expression of *IL1B* and *IL6* mRNA, whereas pre-infection treatment (washed) left it unchanged. No significant change was observed for *CXCL8* mRNA levels upon LF pre-infection treatment (washed), whereas pre-infection treatment (unwashed) significantly upregulated it, as compared to infected untreated control. Moreover, the expression of the potent anti-inflammatory cytokine transforming growth factor beta (*TGFB1*) resulted in being upregulated in infected Caco-2 cells pre-treated (washed and unwashed) with LF, as compared to control (Figure 4B), whereas anti-inflammatory *IL10* transcripts were downregulated. Overall, these data suggest that LF pre-infection treatments can modulate cytokines expression triggered by SARS-CoV-2 in vitro.

## 4. Discussion

LF is a naturally occurring, non-toxic glycoprotein that is orally available as a nutritional supplement. Its broad-spectrum antiviral as well as immunomodulatory [31] and anti-inflammatory [32] actions may be highly relevant to the pathophysiology of COVID-19. Indeed, many opinion articles have suggested LF as a potential prophylactic and adjunct treatment for SARS-CoV-2 infection [12,28,33,34,35]. In the present study, for the first time, we tested the effects of LF on antiviral immune response gene expression and SARS-CoV-2 infection in vitro. Our data show that LF can boost the antiviral immune response and partially inhibit SARS-CoV-2 infection in a human intestinal epithelial cell line.

LF is thought to exert its biological activities following interaction with specific receptors that have been identified in multiple tissues and cell types, including intestinal, placental, hepatocellular, mammary, respiratory epithelial and lymphocytes [36,37]. Specific intestinal receptors that bind LF could be lactoferrin receptor (LfR) ([38], intelectin-1 (IntL) [39], as well as accessory molecules involved in the TLR4 pathway, including CD14 and LPS binding protein [40]. Receptor binding may evoke signaling systems and pathways involving, amongst the others, nuclear factor κB (NF-κB) and various interferon regulatory factors (IRFs), resulting in the modulation of the antiviral immune response [41]. Among the intrinsic antiviral factors of the host, IFNs are involved in numerous initial responses against viral infections. The induction of antiviral cytokine IFN-α/β expression by LF has been reported previously and it has been involved in inhibition of viral replication in the infected cells [17]. Thus, it is not surprising that also in our model LF treatment of the intestinal epithelial cell line Caco-2 could enhance the expression of *IFNA1* and *IFNB1*. Indeed, LF could also induce the expression of IRF 3 and 7, primary transcriptional factors downstream of MAVS signaling that regulate the type I IFN response after RNA virus infections [42]. Interestingly, LF treatment also resulted in the upregulation of *TLRs* involved in RNA virus recognition such as *TLR3* and *TLR7*. This is of special interest in the context of SARS-CoV-2 infection, since TLR7 has been implicated as an important pattern recognition receptor in the recognition of ssRNA of the Middle East respiratory syndrome coronavirus (MERS-CoV) and severe acute respiratory syndrome CoV (SARS-CoV) in murine infection models, making it a likely candidate to function as a central pattern recognition receptor of SARS-CoV-2 [43,44]. Moreover, a previous study showed that bovine LF supplementation to elderly patients could especially enhance TLR7-mediated responses in plasmacytoid dendritic cells from elderly women, thus suggesting that LF may also contribute to host antiviral responses in vivo [45].

Gastrointestinal symptoms can occur in a broad range of SARS-CoV-2 patients and can sometimes precede the respiratory involvement and be the first manifestation of the infection [46]. Indeed, gut epithelium expresses the ACE2 receptor, recognized by SARS-CoV-2 to enter host cells, where the virus starts to replicate and to elicit immune system activation characterized by pro-inflammatory cytokines and immune cell recruitment [47]. Possibly, the immune response could lead to a dysbiosis with a propagation of the pro-inflammatory state [47]. Following oral administration, abundant LF remains on the lining of the gastrointestinal tract [48], and thus it may be plausible that LF could protect host cells against infection by SARS-CoV-2. LF possesses strong antiviral activity against a broad spectrum of RNA and DNA viruses that typically utilize common molecules on the cell membrane to facilitate their invasion into cells [16]. These molecules, including HSPGs, provide the first anchoring sites on the cell surface and help the virus make primary contact with host cells. LF binding capacity to HSPGs competes with virus for receptor occupancy, therefore it could interfere with virus cell entry. Indeed, it has been shown that LF can block the infection of SARS pseudovirus by binding to HSPGs, thus suggesting that it may also exert a protective role in the host immune defense against SARS-CoV-2 invasion, since HSPGs are also co-factors for SARS-CoV-2 cell entry [5,13]. Moreover, LF strongly inhibits cathepsin L [22], whose inhibition by the specific inhibitor SID 26,681,509 was shown to block SARS-CoV-2 entry into human embryonic kidney 293/hACE2 cells [23]. In our model, LF had a modest but significant antiviral activity against SARS-CoV-2 infection, suggesting that potential LF-mediated HSPG blocking or cathepsin L inhibition may not be sufficient for the complete inhibition of SARS-CoV-2 infection. Moreover, LF showed the same antiviral activity in the pre-infection treatments (washed and unwashed) protocols, suggesting that its effect is mainly mediated by interaction with the host cell rather than with the virus itself.

A limitation of our in vitro study was the lack of use of conventional antiviral drugs to compare the antiviral effectivity of LF and of blocking assays to confirm the actual antiviral action of LF. Indeed, the detailed molecular mechanisms by which LF inhibits SARS-CoV-2 infection in vitro remain an issue to be solved. On the other hand, our promising results may justify the investigation of LF in combination with other nutraceuticals to verify their possible synergistic effects. In particular, LF has been shown to increase the expression of vitamin D receptor (VDR) in the colon of vitamin D deficient mice [49] and a growing body of evidence suggests that vitamin D deficiency may play a central role in COVID-19 pathophysiology and mortality [50,51]. Thus, LF and vitamin D combined supplementation may represent a valid adjuvant therapeutic tool in patients with COVID-19 in light of their well-known anti-inflammatory and immunomodulatory properties [52,53,54].

Current thinking suggests that mortality from COVID-19 is not simply due to viral infection but is the result of a cytokine storm syndrome in a subgroup of patients associated with hyperinflammation leading to acute respiratory distress and subsequent mortality [27]. Notably, in our experimental setting LF pre-infection treatments (washed and unwashed) of SARS-CoV-2 infected cells could induce the expression of *TGFB1,* an immune suppressive cytokine pivotal for immune responses regulation, and dampen the expression of *TSLP*, which plays a critical role in initiation and perpetuation of airway inflammation by activating inflammatory and smooth muscle cells [55] and has been shown to be deregulated in intestinal inflammatory disorders such as ulcerative colitis where it induces the T helper 2 component [56]. By contrast, LF treatment decreased the anti-inflammatory IL10 transcripts level. However, its expression was just modestly affected by SARS-CoV-2 infection in comparison to the other cytokines (see Supplementary Figure S1). Moreover, although IL10 is most commonly recognized as an anti-inflammatory cytokine, evidence has linked IL10 to a potential role in fibrosis, where increased IL10 expression was reported to induce collagen production and fibrocyte recruitment into the lung [57]. Notably, LF pre-infection treatment (unwashed) resulted in decreased expression of *IL1B* and *IL6* transcripts, proinflammatory cytokines associated to the cytokine storm [27]. These immunomodulatory effects of LF might counteract the activation of the cytokine-storm, thus protecting from COVID-19 exacerbation.

As there is currently just one conditionally authorized anti-viral treatment regimen for COVID-19 and two vaccines approved for emergency use against SARS-CoV-2 infection [10], one could contemplate the use of LF as a non-toxic health supplement. Our in vitro data support LF as an immune booster of the antiviral immune response with moderate effects against SARS-CoV-2 infection in intestinal epithelial cells. Although our findings cannot be directly translated into clinical practice, they should prompt the planning of clinical studies aimed at investigating the potential anti-SARS-CoV-2 activity of LF in vivo. Further experiments are required to elucidate in vivo dosage and efficacy and to clarify whether LF adjuvant treatment could be useful particularly for COVID-19 cases characterized by mild to moderate or severe gastrointestinal GI symptoms.

## Figures and Tables

**Table 1:** Primers used in the study.

Gene	5′-->3′ forward Sequence	5′-->3′ Reverse Sequence	T_a_ °C
*GAPDH*	GACACCCACTCCTCCACCTTT	TTGCTGTAGCCAAATTCGTTGT	60
*IFNA1*	TTCAGGGGCATCAGTCCCTA	CCGTCCATTCCTTGATTTGGTT	60
*IFNB1*	TCTCCTGTTGTGCTTCTCCAC	GCCTCCCATTCAATTGCCAC	60
*IL1B*	CTGAGCTCGCCAGTGAAATG	TGTCCATGGCCACAACAACT	60
*IL6*	GTCCAGTTGCCTTCTCCCTGG	CCCATGCTACATTTGCCGAAG	60
*IL10*	GTGAAAACAAGAGCAAGGCCG	TAGAGTCGCCACCCTGATGT	60
*TGFB1*	ACTGCGGATCTCTGTGTCAT	AGAGTCCCTGCATCTCAGAGT	60
*CXCL8*	TGGACCCCAAGGAAAACTGG	ATTTGCTTGAAGTTTCACTGGCA	60
*TSLP*	AAGGCAACAGCATGGGTGAA	GGGAACATACGTGGACACCC	60
*TLR3*	CCTTTTGCCCTTTGGGATGC	TGAAGTTGGCGGCTGGTAAT	60
*TLR7*	CCTTGTGCGCCGTGTAAAAA	GGGCACATGCTGAAGAGAGT	60
*MAVS*	GCAATGCCGTTTGCTGAAGA	CGCCGCTGAAGGGTATTGAA	60
*IFIH1*	GCATATGCGCTTTCCCAGTG	CTCTCATCAGCTCTGGCTCG	60
*IRF3*	GAGCTGTGCTGGCGAGAAG	CTCTCCAGGAGCCTTGGTTG	60
*IRF7*	CCATCGGCTTTTGGGTCTGT	TTCCCATGGTCCGGCCTC	60
*CoVE*	ACAGGTACGTTAATAGTTAATAGCGT	ATATTGCAGCAGTACGCACACA	60
*RdRp IP2*	ATGAGCTTAGTCCTGTTG	CTCCCTTTGTTGTGTTGT	60

## Data Availability

The data presented in this study are available on request from the correspondence author.

## Supplementary Materials

The following are available online at https://www.mdpi.com/2072-6643/13/2/328/s1, Figure S1: Modulation of cytokines expression by SARS-CoV-2 infection in vitro.
