# Supplementary material for: Protective Effects of Lactoferrin against SARS-CoV-2 Infection In Vitro
Source: Nutrients. 2021 Jan 23;13(2):328. doi: 10.3390/nu13020328 (PMC7911668; doi:10.3390/nu13020328)
Supplement: Supplementary file 1 [file nutrients-13-00328-s001.pdf]

A

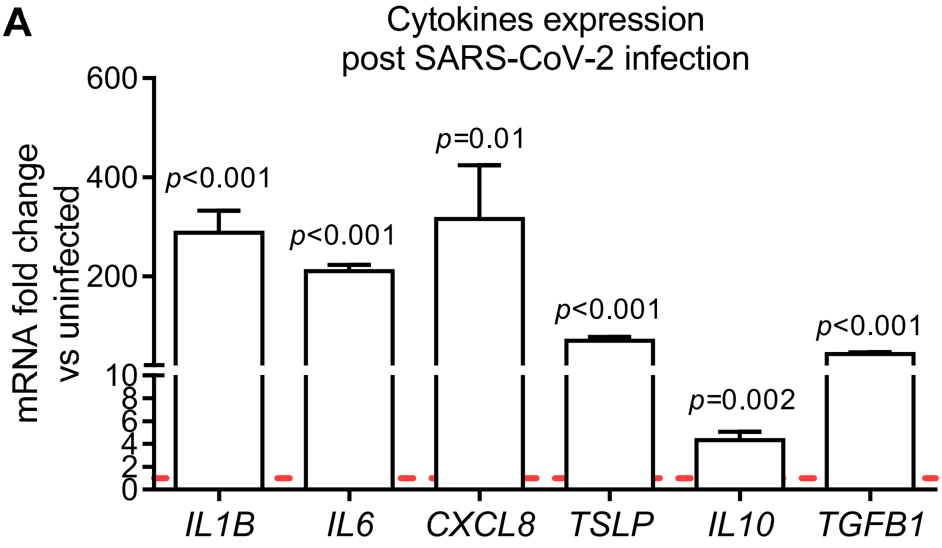

Scheme 1. Modulation of cytokine expression by SARS-CoV-2 infection *in vitro*. (**A**) Caco-2 cells were infected with SARS-CoV-2. Cytokines expression was assessed by real-time qPCR 24 hours post infection (n=3-6). Data are shown as relative fold changes compared to uninfected control (not shown, arbitrarily set as 1) and presented as mean + standard error of the mean.
